# Gout in Pregnancy: A Rare Phenomenon

**DOI:** 10.7759/cureus.11697

**Published:** 2020-11-25

**Authors:** Kevin Pierre, Noah F Gomez, Catarina Canha, Ghania Masri

**Affiliations:** 1 Neurosurgery, University of Florida College of Medicine, Gainesville, USA; 2 Obstetrics and Gynecology, University of Florida College of Medicine, Gainesville, USA; 3 Internal Medicine, University of Florida College of Medicine, Gainesville, USA; 4 Internal Medicine, University of Florida College of Medicine, Jacksonville, USA

**Keywords:** pregnancy, arthritis, sepsis, missed abortion, gout

## Abstract

Gout is a rare phenomenon in reproductive age women due to the uricosuric effects of estrogen. Furthermore, gout in pregnancy has been reported in only a scant number of case reports. We present the case of a 21-year-old gravida-4 para-3 female at four-weeks gestation presenting with acute polyarticular gout, complicated by Haemophilus influenzae sepsis and hyperemesis gravidarum. The gout flare was likely precipitated by primary H. influenzae bacteremia, which was successfully treated with intravenous antibiotics. The treatment of gout in pregnancy is challenging because of the limited number of guidelines. We demonstrate successful management of an acute gout flare in pregnancy with colchicine and steroid injection of the affected joint. Unfortunately, the patient suffered a miscarriage, but the link between gout flare and spontaneous abortion is tenuous.

## Introduction

Gout is a rheumatologic condition characterized by exquisitely painful arthritis secondary to uric acid crystal deposition. Gout is rare in reproductive age women due to the uricosuric effects of estrogen [1,2]. In pregnant women, gout is exceptionally uncommon, with few reported cases [3,4]. The dearth of case reports presents a challenge for both accurate diagnosis and optimal management of this debilitating disease. We present the case of a 21-year-old multipara with polyarticular gout in the first trimester of pregnancy.

## Case presentation

A 21-year-old gravida-4 para-3 female at four weeks gestation by last menstrual period with a history of hyperemesis gravidarum presented with a four-day history of joint pain, fevers, urinary frequency, and profuse vomiting, associated with dizziness and palpitations. Physical examination was significant for bilateral knee, feet, and shoulder pain, with associated lumbar and right upper quadrant abdominal tenderness, and negative for costovertebral angle tenderness. She was distressed, febrile (39.6°C), and tachypneic (27/min), with tachycardia (129 BPM) unresponsive to intravenous fluid resuscitation. Laboratory studies revealed leukocytosis (13.6 × 103/µL) with a neutrophilic predominance and, hyponatremia, hypokalemia, and widened anion gap metabolic acidosis. Furthermore, the HIV test was negative. Reflex urine culture following a concerning urinalysis revealed Escherichia coli. Blood cultures revealed Haemophilus influenzae (H. influenzae) bacteremia. Transvaginal ultrasound was significant for a gestational sac, yolk sac, and questionable fetal pole, and crown-rump length of 2.2 mm. The definite fetal cardiac activity could not be appreciated.

Twenty-four hours later, she developed swelling of the left knee and right metatarsophalangeal joints. Knee arthrocentesis was significant for monosodium urate crystals, leukocytosis with neutrophilic predominance, and negative for bacterial growth. Of note, serum uric acid studies showed hypouricemia.

Her gout flare resolved with methylprednisolone acetate injection of the left knee and colchicine, which were selected because of low teratogenicity [5]. Hyperemesis gravidarum and metabolic derangements were treated with pyridoxine, promethazine, folic acid, and potassium replacement. Sepsis was likely from primary bacteremia as a workup for known sources of H. influenzae, such as with visualization of the gallbladder via right-upper quadrant ultrasound, was negative [6]. Bacteremia secondary to septic arthritis could not be definitively ruled out considering the administration of antibiotics two days before the diagnostic arthrocentesis. Follow-up blood cultures 72 hours after initiating IV ceftriaxone were negative. She was then treated with IV ceftriaxone for two weeks following discharge. Follow-up transvaginal ultrasound at 10 weeks gestation revealed a missed abortion that was managed with dilation and curettage.

## Discussion

Our patient suffered an episode of primary gout in the first trimester of pregnancy with no predisposing factors, likely precipitated by H. influenzae sepsis with dehydration secondary to sepsis-related vomiting and hyperemesis gravidarum diagnosed this pregnancy [7]. Gout is rare in women of reproductive age, particularly during pregnancy, due to the estrogen’s uricosuric effects [1,2]. The few case reports available are analyzed in two literature reviews, the most recent identifying only 12 women during 23 pregnancies who experienced gout [3,4]. Many of these patients had a history of gout, were diagnosed during previous pregnancies, or were symptomatic postpartum, likely from estrogen withdrawal. In comparison, our patient was diagnosed with gout after having gout flare antepartum. Furthermore, she was also much younger, at the age of 21 years, compared to the average age of 30.6 (±3.69) in previous case reports.

Whereas our patient had low serum uric acid levels, pregnant patients with gout generally have elevated uric acid levels [3]. However, low uric acid levels are known to occur during acute gout attacks [8]. Furthermore, some patients had a family history of gout, but our patient did not [3]. Alterations in renal excretion of uric acid may be due to decreasing glomerular filtration [9]. For example, most patients with acute gout attacks during their pregnancies had chronic renal failure. Our patient showed no evidence of kidney dysfunction. Repeat transvaginal ultrasound approximately one month after discharge revealed only a gestational sac with an irregularly shaped yolk sac. Unfortunately, the patient was diagnosed with a failed early intrauterine pregnancy. Case reports suggest an association between gout in pregnancy and spontaneous abortion; however, these cases are complicated by comorbidities such as renal failure from superimposed preeclampsia, which are also risk factors for intrauterine fetal demise [3,4]. Lastly, cases showed that pregnant patients with gout were likely to have maternal complications [3]. It is unclear whether there was an association between our patient’s gout and her presenting comorbidities.

The viability of the patient’s pregnancy on initial presentation is debatable due to the absence of definitive cardiac activity in the setting of a present yolk and gestational sac. An abnormal pregnancy would have likely precluded uricosuric estrogen levels sufficient to prevent hyperuricemia. If this were the case, her gout flare may have in fact resulted exclusively from H. influenzae sepsis and associated dehydration.

## Conclusions

While rare in pregnancy, gout should be suspected when a patient presents with characteristic symptoms like acute onset arthritis with swelling and warmth. Treatment is challenging due to the limited information on the safety of gout drugs in pregnancy, considering the rarity of this occurrence. Treatment should focus on maintaining renal function, managing comorbidities, and treating and preventing flares during the pregnancy, with supportive measures given before pharmacological treatments. Pharmacological treatment should be based on risk assessments according to the patient’s gestational age and comorbidities.
